# Global prevalence of musculoskeletal symptoms among restaurant workers: A systematic review and meta-analysis

**DOI:** 10.1016/j.puhip.2026.100750

**Published:** 2026-02-12

**Authors:** Faezeh Makki, Javad Koroujde, Anahita Hejazi, Ali Sahebi, Mohammad Aref Rajaee, Somayeh Tahernejad

**Affiliations:** a Health in Disasters and Emergencies Research Center, Institute for Futures Studies in Health, Kerman University of Medical Sciences, Kerman, Iran; b MSc, HSE Sirjan Golgohar Organization, Kerman, Iran; c Non-Communicable Diseases Research Center, Ilam University of Medical Sciences, Ilam, Iran; d Department of Medical Emergencies and Health in Disasters and Emergencies, Ilam University of Medical Sciences, Ilam, Iran

**Keywords:** Musculoskeletal disorders, Restaurant worker, Risk factors, Prevention, Ergonomics

## Abstract

**Objectives:**

To gain a comprehensive understanding of the prevalence of MSSs among restaurant workers, a systematic review and meta-analysis was conducted. The primary objective was to determine the magnitude of this issue and to provide valuable insights for the development and implementation of effective prevention and intervention strategies.

**Study design:**

systematic review and meta-analysis.

**Methods:**

This study adhered to the Preferred Reporting Items for Systematic Reviews and Meta-Analyses (PRISMA) guidelines for systematic reviews and meta-analyses. Relevant studies were identified through a comprehensive search of multiple databases, including PubMed, Scopus, Web of Science, and Google Scholar. A random-effects model was employed for the meta-analysis, and heterogeneity among studies was assessed using the I^2^ statistic. Data analysis was conducted using STATA statistical software (version 14).

**Results:**

Following the completing the stages of screening, selection, and evaluation of the quality of the studies, 17 studies were included in the meta-analysis. The results of the meta-analysis showed that The pooled prevalence of MSSs among restaurant workers was 67.01% (95% CI: 57.90-76.12, I2 = 98.2%, P < 0.001). The highest and lowest prevalence rates are related to the low back with a rate of 50.78% (95% CI: 39.45-62.11, I2 = 98.4%, P < 0.001) and hip/thigh with a rate of 19.05% (95% CI: 10.24-27.87, I2 = 98.2%, P < 0.001).

**Conclusions:**

The relatively high occurrence of MSSs within the restaurant industry necessitates the implementation of preventive measures, including ergonomic interventions, to mitigate their adverse effects.

## 1 Introduction

Work-related musculoskeletal symptoms (MSSs) present a major challenge for countless employees around the globe [1]. Studies repeatedly show that these symptoms are the second most common reason for disability worldwide [2,3]. They not only interfere with physical functioning but also greatly affect the overall health of workers, frequently resulting in reduced productivity. Identifying the exact musculoskeletal issues linked to a specific job can be challenging. Nonetheless, a variety of research has highlighted that the ergonomic risk factors associated with different professions can greatly heighten the probability of experiencing or exacerbating these conditions [4].

The nature of the job in the HORECA (hotels, restaurants and catering) sector can be challenging, as it involves high job demands such as direct customer interaction, high physical workloads, and irregular working hours. As a result, there is a significant risk of work-related health issues, particularly musculoskeletal disorders [5].

Restaurant workers are at high risk for musculoskeletal problems even though their work relies heavily on the musculoskeletal system [[6], [7], [8], [9]]. During their work, restaurant workers are often exposed to significant physical and mechanical risk factors, which are one of the main reasons for creating or worsening MSSs. These risk factors include long working hours, static working postures, frequent bending of the trunk, repetitive upper limb movements, prolonged standing, and lifting heavy loads in improper postures [8,10].

Several research studies have investigated the frequency of MSSs among restaurant workers. A research study on the working posture of restaurant chefs found that the most common areas for painful working postures were the neck and wrist, which had the highest reported level of risk [11]. In the same way, by examining the working posture of the upper limbs of restaurant workers, another study reported that the highest scores were related to the tasks of cleaning the kitchen, carrying the pot, and pouring ingredients, respectively. This study also revealed that the highest average scores for each of the examined body parts were obtained for the neck, wrist, and trunk, respectively [12]. Additionally, research revealed that Chinese cooks face a 1.29 to 1.35 times higher risk of MSSs compared to their counterparts [9]. In another study, the highest prevalence of MSSs was reported to be related to the back and knees [13].

In a study, a significant correlation was found between the lack of adequate rest and a higher likelihood of MSSs in female kitchen workers [10]. An intervention study also reported that the implementation of an educational intervention led to a significant improvement in workers' knowledge and performance regarding work-related musculoskeletal disorders (WMSDs). This improvement was observed after the intervention [14]. Based on the literature, it appears that employees in the restaurant industry are vulnerable to MSSs, which can ultimately impact their performance and ability to work effectively. The prevalence of MSSs among restaurant workers has been a concern, and studies show that these disorders are common and can lead to chefs leaving their jobs [15]. In recent times, a significant amount of research has come to light that looks into how common MSSs are among restaurant staff in various communities. However, to our knowledge, despite these efforts, there hasn't been a thorough investigation that systematically assesses the overall occurrence of MSSs in this particular occupation. Acknowledging the profound effect of MSSs on the well-being of restaurant employees and the urgent necessity to fill this knowledge void, we initiated a systematic review and meta-analysis. The main goal of this research is to attain a detailed understanding of how widespread MSSs are among restaurant workers. We anticipate that the results of this study will offer valuable information for those managing occupational health, aiding them in creating specialized training programs and adopting effective ergonomic measures to reduce the risk of MSSs in this workforce.

## 2 Methods

### 2.1 Study design: systematic review and meta-analysis

The current research adhered to the Preferred Reporting Items for Systematic Reviews and Meta-Analyses (PRISMA) guidelines [16]. This commitment helped us uphold clarity and strong methodology throughout the review process. To enhance the trustworthiness and reproducibility of our findings, we proactively registered our study protocol with the International Prospective Register of Systematic Reviews (PROSPERO), using the registration number CDR42024507102. This registration offers a publicly available account of our intended methods, helping to reduce potential biases and uphold the integrity of the research.

Following the PRISMA protocol closely, we adopted a systematic and thorough approach in every phase of this review. This involved creating and implementing a detailed search strategy to locate all relevant studies, followed by a careful screening process to filter out irrelevant research and focus on those that met our inclusion criteria. Two independent researchers then undertook the tasks of selecting studies, assessing quality, and extracting data. To reduce subjective bias and improve the reliability of the data extraction, the two researchers worked separately at first. Any differences that arose were resolved through collective discussions, allowing for a meticulous review of the data and reaching a consensus. This iterative approach guaranteed the precision and uniformity of the data collected, establishing a robust basis for further analyses [17].

### 2.2 Information sources and search strategy

A thorough approach was taken to uncover pertinent literature. Several electronic databases were methodically explored, including PubMed, Scopus, Web of Science, Science Direct, and Google Scholar. To guarantee a thorough investigation, this process was enhanced by manually reviewing conference proceedings, the reference lists of selected studies, and existing systematic reviews. To refine the search terms, pertinent keywords were extracted from diverse sources, such as the Medical Subject Headings (MeSH) thesaurus, keywords from related articles, and conversations with subject matter experts.

The search strategies tailored for each database were carefully crafted, incorporating a mix of relevant keywords such as: “Restaurant chef∗”, “Restaurant worker∗”, “Male kitchen workers∗”, “Restaurant sector∗”, “Food stall workers∗”, “Musculoskeletal complaint∗”, “WRMSDs”, “Work related musculoskeletal disorder∗”, “MSDs”, “MSSs”, “Muscle problem∗”, “Muscle strain∗”, “Musculoskeletal disease∗”, “Musculoskeletal symptom∗”, “Musculoskeletal problem∗”, “Musculoskeletal disorder∗”, “Musculoskeletal pain”, “Back pain∗”, “Neck pain∗”, “Dysfunction∗”, “Arthritis joint∗”, “Arthritis bone∗”, “Shoulder pain∗”, “Elbow pain∗”, “Hand pain∗”. These keywords were strategically combined using suitable Boolean operators and searched across different fields within each database. The search continued until the end of May 2023 to include the latest relevant research findings. A comprehensive overview of the search strategy applied across all databases can be found in Table 1.

**Table 1:** Search instructions for different databases types.

Database	Search strategy
Pubmed	((“restaurant chef∗” OR “Restaurant worker∗” OR “male kitchen workers∗” OR “Restaurant sector∗”) AND (“Musculoskeletal complaint∗” OR “WRMSDs” OR “Work related Musculoskeletal disorder∗” OR “MSDs” OR “MSSs” OR “Muscle problem∗” OR “Muscle strain∗” OR “Musculoskeletal disease∗” OR “Musculoskeletal symptom∗” OR “Musculoskeletal problem∗” OR “Musculoskeletal disorder∗” OR “Musculoskeletal pain” OR “Back pain∗” OR “Neck pain∗” OR “Dysfunction∗” OR “Low Back Pain ∗” OR “Arthritis bone∗” OR “Shoulder pain∗” OR “Elbow pain∗” OR “Hand pain∗”))
Scopus	(((ALL(“Restaurant worker∗”) OR ALL(“restaurant chef∗”) OR ALL(“Restaurant sector∗”) OR ALL(“Food stall workers∗”)) AND (ALL(“Musculoskeletal complaint∗”) OR ALL(“WRMSDs”) OR ALL(“Work related Musculoskeletal disorder∗”) OR ALL(“MSDs”) OR ALL(“Muscle problem∗”) OR ALL(“Muscle strain∗”) OR ALL(“Musculoskeletal disease∗”) OR ALL(“Musculoskeletal symptom∗”) OR ALL(“MSSs”) OR ALL(“Musculoskeletal problem∗”) OR ALL(“Musculoskeletal disorder∗”) OR ALL(“Musculoskeletal pain∗”) OR ALL(“Back pain∗”) OR ALL(“Neck pain∗”) OR ALL(“Dysfunction∗”) OR ALL(“Arthritis joint∗”) OR ALL(“Arthritis bone∗”) OR ALL(“Shoulder pain∗”) OR ALL(“Elbow pain∗”) OR ALL(“Hand pain∗”))))
Web Of Science (WOS)	(((TS=(“ Food stall workers∗”) OR TS=(“ Restaurant sector∗”) OR TS=(“ restaurant chef∗”) OR TS=(“ male kitchen workers∗”)) AND (TS=(“Musculoskeletal complaint∗”) OR TS=(“WRMSDs”) OR TS=(“Work related Musculoskeletal disorder∗”) OR TS=(“MSDs”) OR TS=(“MSSs”) OR TS=(“Muscle problem∗”) OR TS=(“Muscle strain∗”) OR TS=(“Musculoskeletal disease”) OR TS=(“Musculoskeletal symptom∗”) OR TS=(“Musculoskeletal problem∗”) OR TS=(“Musculoskeletal disorder∗”) OR TS=(“Musculoskeletal pain∗”) OR TS=(“Back pain∗”) OR TS=(“Neck pain∗”) OR TS=(“Dysfunction∗”) OR TS=(“Arthritis joint∗”) OR TS=(“Arthritis bone∗”) OR TS=(“Shoulder pain∗”) OR TS=(“Elbow pain∗”) OR TS=(“Hand pain∗”))))

### 2.3 Inclusion criteria

The inclusion criteria for this research were meticulously established by applying the PECO (Population, Exposure, Comparison, Outcome) framework, which serves as a structured approach to defining the elements of the study. In this context, the framework specifically represents the population as restaurant workers, the exposure as the experience of working in a restaurant environment, the comparison as any relevant baseline or control group, and the outcome as MSSs.

As a result of this careful application of the PECO framework, every study that mentioned MSSs in relation to restaurant workers was systematically incorporated into the research. This detailed strategy allowed us to gather a diverse range of relevant research, leading to a deeper insight into the frequency and effects of MSSs on those working in the restaurant sector. By applying this structure, we intended to establish a clear focus for our study, which not only made it easier to choose suitable research but also strengthened the overall credibility and reliability of our results.

### 2.4 Exclusion criteria

This comprehensive review set aside several types of research designs that were considered inappropriate for answering the research question. For instance, case reports were omitted since they focus on the experiences of individual patients, making it difficult to generalize the findings. Likewise, while review articles can be useful for compiling existing information, they were not included in this review because they do not offer original primary data. Additionally, studies that examined specific treatments or prevention strategies were excluded, as they did not specifically address the prevalence or incidence of musculoskeletal injuries within the target group. Moreover, research centered on injuries caused by accidents was also left out, since the main emphasis of this review was on musculoskeletal symptoms that occur from regular work activities, rather than those stemming from unforeseen incidents. Lastly, letters to the editor, which typically convey personal opinions or initial observations, were excluded due to their limited methodological strength and insufficient data.

### 2.5 Selection of studies

To effectively manage the identified articles and ensure a streamlined review process, all retrieved records were imported into EndNote X7, a widely used bibliographic management software. This facilitated efficient organization, deduplication, and subsequent stages of the review process. After the initial import, a thorough process to remove duplicates was carried out using EndNote X7 to clear any records that might have been accidently gathered from various databases. Following this, two separate researchers diligently went through the titles and summaries of all the articles left. This first screening step was designed to quickly pinpoint and dismiss articles that obviously did not fulfill the inclusion criteria, thus significantly lowering the number of articles that needed a detailed review. After this preliminary screening, the two researchers performed an in-depth examination of the full texts of all articles deemed potentially relevant. During this phase, each researcher meticulously analyzed the complete text of each article to determine its eligibility according to the established inclusion and exclusion standards. Ultimately, only those articles that clearly satisfied all the inclusion requirements were chosen for the meta-analysis stage of this study.

### 2.6 Qualitative assessment and data extraction

In order to guarantee a thorough evaluation of the quality of studies, two separate reviewers analyzed each study that was included, utilizing the Appraisal tool for Cross-Sectional Studies (AXIS) [18,19]. This validated tool is specifically crafted for evaluating the methodological integrity of cross-sectional research and features a uniform scoring system with a highest possible score of 20 points. Only those studies that obtained a score of 12 or above, reflecting an acceptable standard of methodological rigor, were deemed suitable for inclusion in the meta-analysis [20].

To improve precision and reduce the likelihood of subjective bias, these independent reviewers meticulously gathered essential characteristics from each selected study. This process encompassed details such as the name of the first author, the total sample size, the average age of participants, type of restaurant workers, information regarding the measurement tools utilized for evaluating musculoskeletal symptoms, the gender distribution within the study population, and the incidence of reported musculoskeletal issues. All collected data were systematically documented in a pre-structured and standardized data extraction checklist. This collaborative reviewer method allowed for the identification and resolution of any inconsistencies, thereby ensuring the accuracy and dependability of the gathered information.

### 2.7 Statistical analysis

During this stage, a careful process was initiated to gather essential data from each study that was included. Important details, such as the prevalence of MSSs among restaurant employees and the sample sizes relevant to each study, were diligently extracted and noted. Following this, the variance for each individual study was computed using a binomial distribution, which took into account the natural fluctuations in the reported prevalence of MSSs within each group studied.

To bring together the results from the varied studies included, a weighted average method was utilized to calculate the overall prevalence of MSSs in the entire population examined. This method placed more significance on studies that had larger sample sizes and less variability within them, thus yielding a more accurate and representative estimate of the true prevalence.

In the final analysis, a meta-analysis was performed using a straightforward random-effects model. This model considers both the variability within individual studies and the differences between studies, resulting in a more cautious estimate of the overall effect size. The degree of heterogeneity among the included studies was measured using the I^2^ statistic, a well-established metric that evaluates the level of variability in the effect estimates that goes beyond what could be attributed to chance. Heterogeneity was classified as follows: negligible (<25%), moderate (25-50%), high(50-75%), and very high (>75%) [21].

To assess the potential influence of publication bias, Begg's test was carried out. Publication bias refers to the inclination for studies with statistically significant outcomes to be more frequently published, which can skew the overall results of a meta-analysis. All statistical evaluations were executed using STATA statistical software, version 14, a well-known and reliable statistical tool.

## 3 Results

### 3.1 Systematic review results

**Fig. 1:**
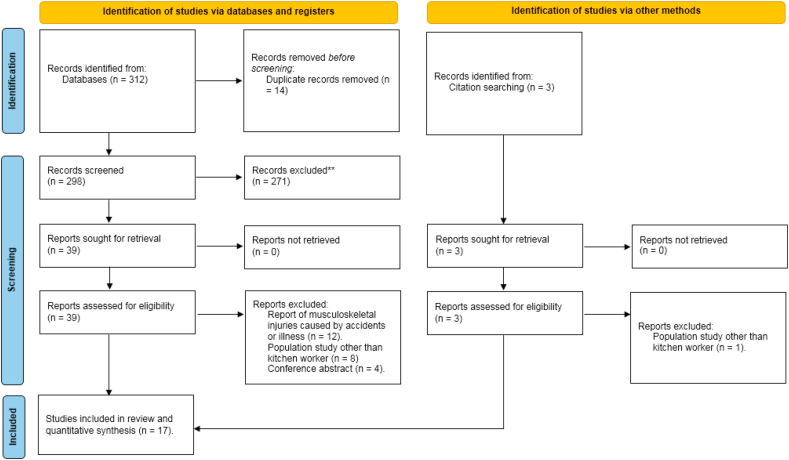
Flowchart of the selection of studies based on PRISMA.

An initial comprehensive search of the electronic databases yielded a substantial number of 312 potentially relevant articles. Following the removal of duplicate records, a total of 298 unique articles proceeded to the subsequent screening stage. A thorough two-phase review process was subsequently carried out. During the initial phase, two independent evaluators carefully examined the titles and abstracts of all 298 articles to pinpoint those that evidently did not fulfill the inclusion requirements. This preliminary assessment led to the elimination of a considerable number of articles, narrowing the selection down to 39 studies for additional analysis. Subsequently, a thorough full-text review was conducted on these 39 potentially eligible studies. Following this in-depth assessment, 17 studies ultimately met all inclusion criteria and were therefore included in the meta-analysis (see Fig. 1).

This thorough review examined a significant group of 5037 restaurant employees. Among the 17 studies incorporated into the meta-analysis, 14 concentrated on the overall frequency of MSSs. A detailed summary of all the studies included in the meta-analysis, along with important characteristics of each study, can be found in Table 2 for further reference.

**Table 2:** Characteristics of the studies included in the meta-analysis.

First author/Year	Country	Sample size	The total prevalence of MSSs	The prevalence of MSSs by anatomical location	Tools^a^	Quality assessment score
Chyuan (2004) [22]	Taiwan	905	84%	Lower back: 52.7 %	Self-administered questionnaire	18
				Shoulder: 57.9 %		
				Knee: 33.9 %		
				Neck: 54.3 %		
				Upper back: 32.7 %		
				Upper arm: 32.3%		
				Lower arm: 27.3%		
				Finger/wrist: 46.5%		
				Upper leg: 22.3%		
				Lower leg: 33.6%		
				Ankle/foot: 42.8%		
Haukka (2006) [23]	Finland	495 female workers	87%	Neck: 71%	Self-administered questionnaire	19
				Lower back: 50%		
				Shoulder: 34%		
				Ankle/foot: 30%		
				Hips: 19%		
				Knees: 29%		
				Forearms/hands: 49%		
				Upper limb: 53%		
				Lower limb: 48%		
Pehkonen (2009) [24]	Finland	376	NR	Shoulder: 34%	Self-administered questionnaire	15

Liu (2011) [25]	Taiwan	765	85.2%	Neck: 59.9%	Self-administered questionnaire	15
				Shoulder: 63.5%		
				Lower back: 56.9%		
				Upper back: 38.9%		
				Elbow: 43.7%		
				Knee: 31.7%		
				Hips/thighs: 19%		
				Ankle/foot: 25.7%		
				Fingers/wrists: 47.5		
Kohan sal (2013) [26]	Iran	21	72.4%	Lower back: 48.2%	Body ness	13
				Upper back: 26%		
				Neck: 34.4%		
Ilban (2013) [27]	Turkey	448	59%	Neck: 13%	NMQ	16
				Shoulder: 7%		
				Upper arm: 1%		
				Forearm: 1%		
				Finger/wrist: 5%		
				Upper back: 5%		
				Lower back: 26%		
				Upper leg: 16%		
				Knee: 13%		
				Lower leg: 17%		
				Ankle/foot: 29%		
Chyuan (2007) [28]	Taiwan	217	NR	Shoulder: 41.1%	NMQ	15
				Hand/wrists: 38.2%		
				Lower back: 40.1%		
Subramaniam (2015) [29]	India	114	67.5%	Neck: 38.6%	NMQ	17
				Shoulder: 62.3%		
				Finger/wrist: 43.9%		
				Elbow/forearm: 31.6%		
				Chest: 20.2%		
				Upper back: 21.1%		
				Lower back: 65.8%		
				Thigh: 30.7%		
				Knee/foot: 42.1%		
Davis (2016) [30]	United States	20	NR	Lower back: 50%	Self-reported questionnaire	18
				Upper back: 55%		
				Neck: 45%		
Elsayed (2017) [14]	Egypt	43	62.8%	Neck: 66.7%	SNQ	17
				Shoulder: 83.3%		
				Upper back: 56.7%		
				Lower back: 93.3%		
				Wrist/hand: 76.7%		
				Elbow: 50%		
				Knee: 60%		
				Hips/thighs: 43.3%		
				Ankle/foot: 53.3%		
Jahangiri (2019) [31]	Iran	300	70%	Back: 57%	Self-reported questionnaire	19
				Knee: 53.3%		
				Neck: 32.7%		
				Shoulder: 16.3%		
				Elbow: 3%		
				Dryness of joint: 4%		
Tan (2020) [32]	Malaysia	126	64.3%	Neck: 26.2%	NMQ	18
				Shoulder: 48.5%		
				Elbow: 19.1%		
				Wrist/hand: 46.8%		
				Lower back: 52.4%		
				Upper back: 10.3%		
				One/both hips/thighs: 10.3%		
				One/both knees: 40.5%		
				One/both ankles/feet: 59.5%		
Choudhary (2020) [33]	Belgium	200	18.5%	Low back: 8.0%	NMQ	14
				Neck: 4.5%		
				Shoulder: 3.5%		
				Wrist/hand: 2.0%		
				Knees: 2.0%		
				Hips/thighs: 1.5%		
				Ankle/foot: 1.0%		
Khutale (2020) [34]	Indian	93	41%	Lower back pain: 41%	NMQ	17
				Knee: 31%		
				Neck: 20%		
				Shoulder: 19%		
				Wrist/hand: 8%		
				Upper back: 6%		
				Ankle/foot: 4%		
Yang (2021) [35]	Chinese	137	67.9%	Neck: 35.8%	Self-administered questionnaire	14
				Shoulder: 35.8%		
Tegenu (2021) [36]	Ethiopia	595 (Female = 419 Male = 176)	81.5%	Lower back: 53.8 %	NMQ	18
				Shoulders: 44.7 %		
				Knee: 40.7 %		
				Neck: 31.6%		
				Upper back: 53.5 %		
				Elbow: 53.8%		
				Ankles: 41.3 %		
				Wrist/hand: 51.6 %		
				Hips/tights: 33.6%		
Ahmad (2023) [37]	Malaysia	191	74.9%	Upper back pain: 55.6%	NMQ	14
				Lower back pain: 73.3%		
				Thighs: 2.6%		
				Feet: 3.1%		

**NR:** Not Reported.

a **NMQ**: Nordic Musculoskeletal Questionnaire, **SNQ**: Standardized Nordic Questionnaire.

### 3.2 Meta-analysis results

**Fig. 2:**
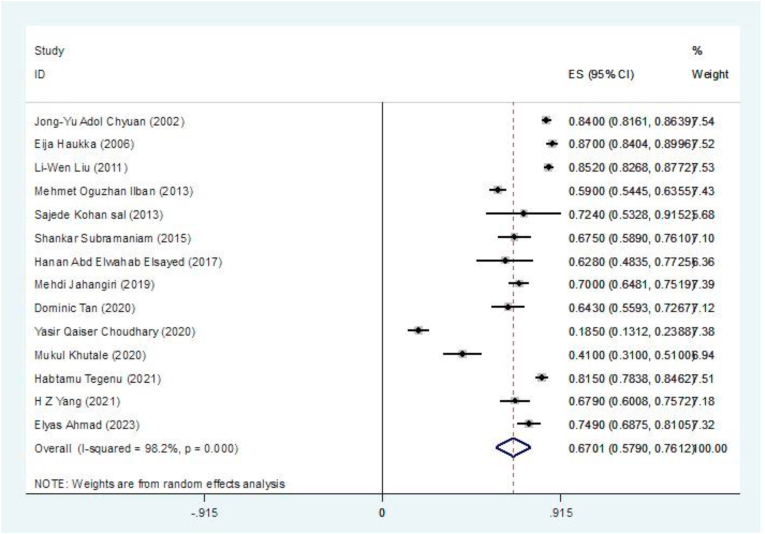
The overall prevalence of MSSs among restaurant workers and the 95% confidence interval for each of the studies reviewed and all studies conducted.

Based on the results of the meta-analysis, the pooled prevalence of MSSs among restaurant workers, defined as experiencing at least minimal pain in any body region, was estimated to be 67.01% (95% CI: 57.90–76.12, I^2^ = 98.2%, P < 0.001). This finding suggests a high prevalence of MSSs within this occupational group. The very high I^2^ index indicates significant heterogeneity among the included studies (Fig. 2). This considerable heterogeneity suggests a wide range in the reported occurrence of MSSs in the various studies, which could be due to differences in study designs, the characteristics of participants, and the specific methods used to evaluate MSSs. To explore the possible effects of publication bias on the overall findings of the meta-analysis, Begg's test was performed. The outcome of Begg's test (P = 0.250) did not indicate any significant publication bias concerning the overall prevalence of MSSs among restaurant employees. This finding suggests that the observed heterogeneity is likely due to factors other than selective reporting of study findings.

**Fig. 3:**
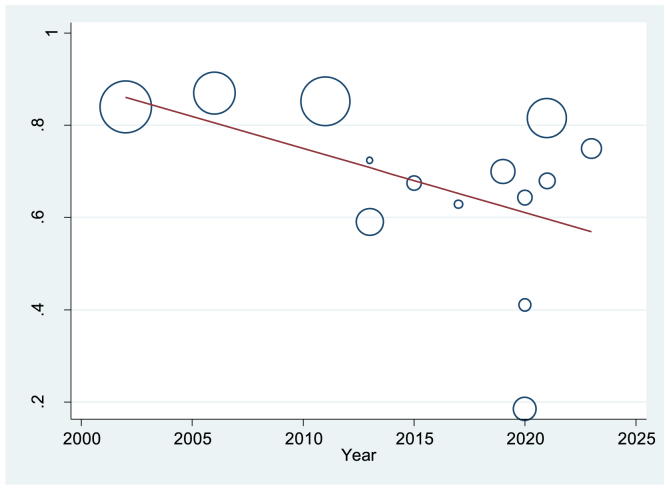
Meta-regression graph of the prevalence of MSSs over time.

The meta-regression results using Egger's test indicated a significant inverse association between the year and the prevalence of MSSs (P < 0.0001), suggesting a steady decline in the prevalence of these symptoms over time (Fig. 3).

Table 3 presents the findings of the subgroup analysis, which aimed to investigate the prevalence of MSSs across different body regions among restaurant workers. This analysis revealed significant variations in the prevalence of MSSs across different body parts. The highest prevalence was observed in the lower back, with an estimated prevalence of 50.78% (95% CI: 39.45–62.11, I^2^ = 98.4%, P < 0.001). In contrast, the lowest prevalence was observed in the hip/thigh region, with an estimated prevalence of 19.05% (95% CI: 10.24–27.87, I^2^ = 98.2%, P < 0.001). These findings suggest that the lower back may be particularly susceptible to musculoskeletal symptoms among restaurant workers.

**Table 3:** Meta-analysis results for various body regions.

MSSs	Number of studies	Sample size	Prevalence of MSSs	95% CI	I^2^	Begg's test	Egger's test
**Lower back**	14	4230	50.78%	39.45% - 62.11%	98.4%	P = 0.702	P = 0.426
**Knee**	11	4081	33.87%	22.85% - 44.89%	98.8%	P = 0.586	P = 0.019∗
**Shoulder**	14	4802	39.17%	26.09% - 52.26%	99.2%	P = 0.352	P = 0.103
**Elbow**	7	2848	32.25%	15.38% - 49.11%	99.2%	P = 0.652	P = 0.210
**Neck**	14	3959	37.97%	24.83% - 51.10%	99%	P = 0.298	P = 0.650
**Upper back**	11	3318	32.20%	19.47% - 44.93%	98.8%	P = 0.815	P = 0.227
**Hand**	11	3998	37.48%	23.02% - 51.94%	99.4%	P = 0.938	P = 0.027
**Hip/Thighs**	8	2529	19.05%	10.24% - 27.87%	98.2%	P = 0.485	P = 0.059
**Foot**	10	3858	28.49%	16.46% - 40.51%	99.3%	P = 0.421	P = 0.014∗

**CI:** Confidence Interval, **I^2^:** I Squared.

The asterisk (∗) indicates a significant difference (P < 0.05).

The I^2^ values presented in Table 3 for each body region demonstrate a very high level of heterogeneity across the included studies. This high degree of heterogeneity indicates substantial variability in the reported prevalence of MSSs across different studies for each body region, potentially attributable to factors such as variations in study design, participant characteristics, and the specific methodologies used to assess MSSs.

To investigate the potential impact of publication bias on the subgroup analysis, Begg's test was conducted for each body region. The results of Begg's test, as presented in Table 3, did not provide evidence of significant publication bias in the reported prevalence of MSSs across any of the examined body regions among restaurant workers.

## 4 Discussion

This study aimed to determine the prevalence of MSSs among restaurant workers through a meta-analysis of 17 studies. The findings revealed an overall MSS prevalence of 67.01% among restaurant employees. Breakdown by body region showed prevalence rates of 50.78% for the lower back, 39.17% for the shoulder, 37.97% for the neck, 37.48% for the wrist/hand, 33.87% for the knee, 32.25% for the elbow, 32.20% for the upper back, 28.49% for the ankles/feet, and 19.05% for the hips/thighs. These results indicate that the lower back is the most commonly affected region.

Among the reviewed studies, four reported an overall prevalence of MSSs exceeding 80%. The study population in the research by Liu et al. was limited to chefs only [25]. The study population in the research by Haukka et al. [23] consisted solely of female restaurant employees. Additionally, the study populations in the research by Tegenu et al. [36] and Chyuan et al. [22] included the entire restaurant staff, comprising chefs, other employees, and service staff. Although their tasks may differ to some extent, in many restaurant work environments, individuals often assist each other in performing various tasks or engage in job rotation. In this study, the number of included studies was limited, precluding the opportunity to conduct subgroup analyses for the different occupations within the restaurant setting.

The majority of studies were conducted in Asian countries. Some studies also investigated ergonomic risk factors in addition to the prevalence of disorders. However, due to the insufficient number of studies or the use of different assessment tools, a systematic review of the ergonomic assessments in this occupational group was not possible.

In a meta-analysis study, the prevalence and incidence of MSSs among European secondary industries were investigated. The food industry was the most prominent studied sector for the prevalence of MSSs subtypes of the back (overall), elbow, leg (overall), shoulder, neck, shoulder/neck, and wrist/hand [38]. Chen et al. found that 93% of bakers experienced musculoskeletal discomfort, and the highest prevalence was in the hand/wrist (right and left) at 66.3% and 51.8%, followed by 50.6% and 45.8% in the shoulders (right and left), and 48.2% below the waist [39]. Tan et al. found that 92.3% of pastry chefs experienced MSSs, with the highest prevalence in the ankles (76.9%), upper back (60.6%), and shoulders (58.7%) [3]. The current research indicates that the prevalence of MSSs in the back is higher than in other areas. This can be attributed to biomechanical risk factors such as prolonged standing, heavy lifting, repetitive tasks at high frequency, and limited opportunities for recovery time, as well as psychosocial risk factors. These findings are consistent with those of previous studies [40]. Hence, the relatively high prevalence of these symptoms among restaurant workers is not far from expected.

Hayes et al. reported that the prevalence of general musculoskeletal pain among dentists ranged from 64% to 93%, with the most common areas for pain being the back (36.3–60.1%) and neck (19.8–85%) [41]. Moderate to high prevalence of MSSs was observed among dentists, and in this occupational group, the back and shoulder areas were more affected compared to other body parts [42]. The results of a meta-analysis conducted among surgeons showed that the prevalence of pain is between 35% and 60% [43]. Tolera et al. conducted a systematic review and meta-analysis and find that the global prevalence of musculoskeletal disorders among sanitation workers is 40.52%. This prevalence was reported to be 43.32% in high-income countries and 38.58% in low-income countries [44]. In a meta-analysis study conducted among nurses, it was found that the most prevalent MSSs were in the low back (62%) followed by the knee (47%) and shoulder (44%) [45]. In a meta-analysis conducted by Sahebi et al., the prevalence of the low back pain among medical emergency personnel was reported to be 50.30% [46]. According to a review study, 76.9% of farmers were found to have musculoskeletal disorders, and the most common pain was in the low back area with a prevalence of 47.8% [47]. The results of a meta-analysis conducted among miners reported the highest prevalence of musculoskeletal disorders in the upper back (50.39%) and the lowest in the knees (16.03) [48]. Compared to the studies mentioned, the prevalence of MSSs among restaurant workers is generally higher than that observed among surgeons and sanitation workers but lower than among dentists. Notably, in all the referenced studies, the highest prevalence of MSSs occurs in the lower back, consistent with the findings of this study among restaurant workers.

Therefore, people who are involved in biomechanical risk factors such as repetitive work and carrying heavy loads are at risk of suffering from musculoskeletal disorders. Risk factors for back pain related to physical work environment include high work speed, repetitive movements, insufficient recovery time, lifting heavy objects, applying excessive hand force, asymmetric body position (dynamic or static), mechanical pressure, vibration (local or whole body) and the low temperature of the environment [[49], [50], [51]] that most of these conditions exist in the working environment of restaurant employees.

In a review study, age, work stress, gender, manual load-carrying process, working environment temperature, smoking habits, and alcohol consumption were reported among the factors of musculoskeletal disorders in restaurant workers [52]. Long-term standing is also one of the risk factors for MSSs among restaurant staff [53]. Several studies have shown that prolonged standing is associated with various health problems, including back pain, lower extremity discomfort, and varicose veins [[54], [55], [56], [57], [58]]. It has also been found that various undesirable and non-ergonomic tools in each task affect the health of individuals [59]. In various studies, a strong correlation has been observed between heavy load handling and the development of several types of MSSs [[60], [61], [62]]. In this regard, Claudon et al. found that a sharper knife blade reduces muscle activity in the hand and radial deviation of the wrist [63]. In addition, insufficient rest is another reason for the increased risk of MSSs among restaurant staff [10]. It is worth mentioning that psychosocial risk factors can also be effective in the occurrence of MSSs or its exacerbation [64,65]. Work duration on working days is a factor that can be considered a stress predictor in chefs [66]. Additionally, depression resulting from excessive workload and exposure to offensive behaviors in the kitchen is identified as a psychosocial factor within this occupational group [67,68], which can contribute to the development of MSSs.

The widespread occurrence and multifaceted nature of MSSs in restaurant employees highlight the urgency for effective preventive measures. The results of this research can offer essential insights for workplace health managers in crafting strategies to alleviate the impact of MSSs on this group of workers.

### 4.1 Limitation

In the present study, there was heterogeneity among the included studies, which may be related to the sample sizes, instruments, and different cut-off points used in the original studies. In addition, since MSSs were not reported based on gender in the original studies, it was not possible to report MSSs by gender in this study either. Another limitation of this study was that the age of the participants was not considered, as the age of the individuals was not reported in all the primary studies. Furthermore, it was not possible to perform a tool-based subgroup analysis, which was also due to the small number of tools used to examine MSSs. Finally, since the type of MSSs (discomfort, pain, functional limitation) was not reported in the original studies, meta-analysis for these outcomes was not feasible.

### 4.2 Conclusion

The meta-analysis revealed a high overall prevalence of MSSs among restaurant workers, reaching 67.01%. Notably, the lower back and shoulder regions were the most frequently affected areas. Given the significant impact of MSSs on this occupational group, including potential negative effects on work ability, it is crucial to implement effective preventive measures. Such measures could conducting ergonomic assessments of the work environment and employees' workstations, tasks, and tools, designing ergonomic interventions such as ergonomic workplace design, training in ergonomics for food industries, providing training on proper postural behaviors with a focus on the low back and shoulder regions, training in appropriate manual handling of loads, provision of assistive tools for load handling, and incorporating regular rest periods and also standard working hours, appears to be essential for reducing the occurrence of these disorders among restaurant workers.

## Ethics statement and consent to participate

Not applicable.

## Consent for publication

Not applicable.

## Authors' contribution statement

FM and ST contributed to the design, implementation and writing of the research, AS and AH contributed to the analysis of the results, and MR and JK contributed to the implementation of the study. The authors read and approved the final version of the manuscript.

## Availability of data and materials

This study is a systematic review and a meta-analysis. all the data sourced from the articles listed in the tables within the manuscript.

## Clinical trial number

Not applicable.

## Funding

This research received no external funding.

## Declaration of competing interest

We would like to bring to the Editor's attention the following points, which may be regarded as potential conflicts of interest or significant financial involvement in this work.

We hereby confirm that no known conflicts of interest are associated with this publication, and no substantial financial support has been provided for this work that could have influenced its outcomes.

We affirm that the manuscript has been reviewed and approved by all listed authors, and there are no individuals who meet the authorship criteria but are not included in the list. Additionally, we confirm that the order of authors listed in the manuscript has been agreed upon by all of us.

We further confirm that we have taken appropriate measures to safeguard the intellectual property related to this work and that there are no obstacles to its publication, including timing, concerning intellectual property matters. In doing so, we affirm that we have adhered to our institutions’ policies on intellectual property.

We acknowledge that the Corresponding Author serves as the primary contact for the editorial process, including interactions with Editorial Manager and direct communications with the journal office. The Corresponding Author is responsible for keeping co-authors informed about progress, submitting revisions, and approving final proofs. We confirm that we have provided an accurate and up-to-date email address accessible to the Corresponding Author.
